# A [Third] Analysis of Cases of Tetanus Treated in Home Hospitals (during August, September, and October, 1916)

**Published:** 1918-01

**Authors:** 


***A [Third] Analysis of Cases of Tetanus Treated in Home Hospitals (during August, September, and October, 1916).*** By Sir David BRUCE. Abs. from the *Lancet*, June 30, 1917.

B. in the present report examines statistically 200 cases. There was a mortality of 36.5 o/o.

B. states that no additional light is thrown upon the disputed points of the curative action of antitoxin. There is, however, even less evidence than before of the superiority of the intrathecal route, and no indication of any advance in the therapeutic use of serum.

The marked improvement in the general mortality, is probably due to the more careful preventive treatment now given universally. B. repeats his recommendations for immediate and sufficient preventive and curative treatment.

